# Neural substrates of cough control during coughing

**DOI:** 10.1038/s41598-024-51477-x

**Published:** 2024-01-08

**Authors:** Takafumi Sugi, Tomoo Inubushi, Tomohisa Ohno, Yuya Onishi, Takashi Isobe, Takashi Shigematsu, Satoshi Hanai, Yoshiro Okada, Ryosuke Takahashi, Yuichi Tawara, Chie Suzuki, Toshihiko Kanno, Yasuhiro Magata, Ichiro Fujishima, Etsuji Yoshikawa, Yasuomi Ouchi

**Affiliations:** 1 Department of Biofunctional Imaging, Hamamatsu University School of Medicine, 1-20-1, Handayama, Higashi-ku, Hamamatsu, Shizuoka 431-3192 Japan; 2 Department of Rehabilitation Medicine, Hamamatsu City Rehabilitation Hospital, 1-6-1 Wagokita, Naka-ku, Hamamatsu, Shizuoka 433-8511 Japan; 3 Central Research Laboratory, Hamamatsu Photonics K.K., 5000, Hirakuchi, Hamakita-ku, Hamamatsu, Shizuoka 434-8601 Japan; 4 Department of Dentistry, Hamamatsu City Rehabilitation Hospital, 1-6-1 Wagokita, Naka-ku, Hamamatsu, Shizuoka 433-8511 Japan; 5 Department of Rehabilitation, Hamamatsu City Rehabilitation Hospital, 1-6-1 Wagokita, Naka-ku, Hamamatsu, Shizuoka 433-8511 Japan; 6 School of Rehabilitation Sciences, Seirei Christopher University, 3453, Mikatahara, Kita-ku, Hamamatsu, Shizuoka 433-8105 Japan; 7 Department of Molecular Imaging, Hamamatsu University School of Medicine, 1-20-1 Handayama, Higashi-ku, Hamamatsu, Shizuoka 431-3192 Japan; 8 Hamamatsu Medical Imaging Center, Hamamatsu Medical Photonics Foundation, Shizuoka, 434-0041 Japan

**Keywords:** Neuroscience, Psychology, Medical research, Neurology

## Abstract

Cough is known as a protective reflex to keep the airway free from harmful substances. Although brain activity during cough was previously examined mainly by functional magnetic resonance imaging (fMRI) with model analysis, this method does not capture real brain activity during cough. To obtain accurate measurements of brain activity during cough, we conducted whole-brain scans during different coughing tasks while correcting for head motion using a restraint-free positron emission tomography (PET) system. Twenty-four healthy right-handed males underwent multiple PET scans with [^15^O]H_2_O. Four tasks were performed during scans: “resting”; “voluntary cough (VC)”, which simply repeated spontaneous coughing; “induced cough (IC)”, where participants coughed in response to an acid stimulus in the cough‐inducing method with tartaric acid (CiTA); and “suppressed cough (SC)”, where coughing was suppressed against CiTA. The whole brain analyses of motion-corrected data revealed that VC chiefly activated the cerebellum extending to pons. In contrast, CiTA-related tasks (IC and SC) activated the higher sensory regions of the cerebral cortex and associated brain regions. The present results suggest that brain activity during simple cough is controlled chiefly by infratentorial areas, whereas manipulating cough predominantly requires the higher sensory brain regions to allow top-down control of information from the periphery.

## Introduction

Coughing is a protective reflex that protects the airways from harmful substances and is necessary to maintain normal airway patency^1^. Cough consists of inspiration (inspiratory phase), glottal closure and forced expiratory effort (compressive phase), and glottal opening and expiration (expiratory phase). Two types of coughs can be distinguished: voluntary cough (VC), which occurs spontaneously, and induced cough (IC), which occurs reflexively in response to airway stimuli. IC is evoked by mechanical or chemical stimuli applied to the airways, including TRPV-1 agonists such as capsaicin as well as acids, histamine, and other compounds^1–3^. Despite this concept, VC and IC cannot be isolated alone as single components of cough, and IC may have an impulse component to cough.

Cough reflex tests have been used to screen for dysphagia, and capsaicin, citric acid, and tartaric acid have been used as cough inducers in the clinical setting^4–6^. Applying cough induction techniques to dysphagic patients with impaired cough reflexes is an important tool to prevent aspiration. Recently, we found tartaric acid to be useful in cough induction for dysphagic patients with silent aspiration; this method was named the cough‐inducing method with tartaric acid (CiTA)^7^. Hence, clinical experience also emphasizes the contribution of the brain to cough regulation.

Brain activity related to cough has been evaluated by a model-based fMRI technique as follows: VC activates a wide range of brain regions, such as the sensorimotor, supplementary motor, orbitofrontal, insular and middle cingulate cortices, thalamus, caudate nucleus, putamen and cerebellum^8,9^. IC also activates the widely distributed brain network, including the somatosensory, insular, middle cingulate, orbitofrontal and supplementary motor cortices, medulla oblongata, and cerebellum^9,10^. Suppression of cough(SC) is regulated by the middle cingulate, supplementary motor and insular cortices, right inferior frontal gyrus, caudate nucleus^9,11^. Thus, many brain regions are believed to play important roles in cough control through a top-down system, in which higher brain regions send information to the brainstem^9,12^. In addition, brainstem activity in the solitary nucleus, paratrigeminal nucleus, trigeminal nucleus^13^ is also implicated in cough induction. These findings were generated using a sophisticated model-based analysis to eliminate artifacts of cough-related head motion.

A major issue in imaging studies of brain activity during cough is the presence of artifacts caused by head movement. Despite fMRI reports using sophisticated methods such as modeling physiological factors during tasks and correcting head movement computationally^14,15^, no real brain activity occurring during cough can be successfully captured with existing techniques. To address this limitation, we have recently developed a PET camera equipped with a free-moving function that enables imaging during head motion^16,17^. The purpose of the present research was to evaluate brain activity changes during different coughing tasks in normal subjects using this restraint-free PET system to clarify the neurophysiology of cough. We hypothesized that brain activity during cough would be more localized than previously reported.

## Results

### Physiological data during PET measurement

**Figure 1:**
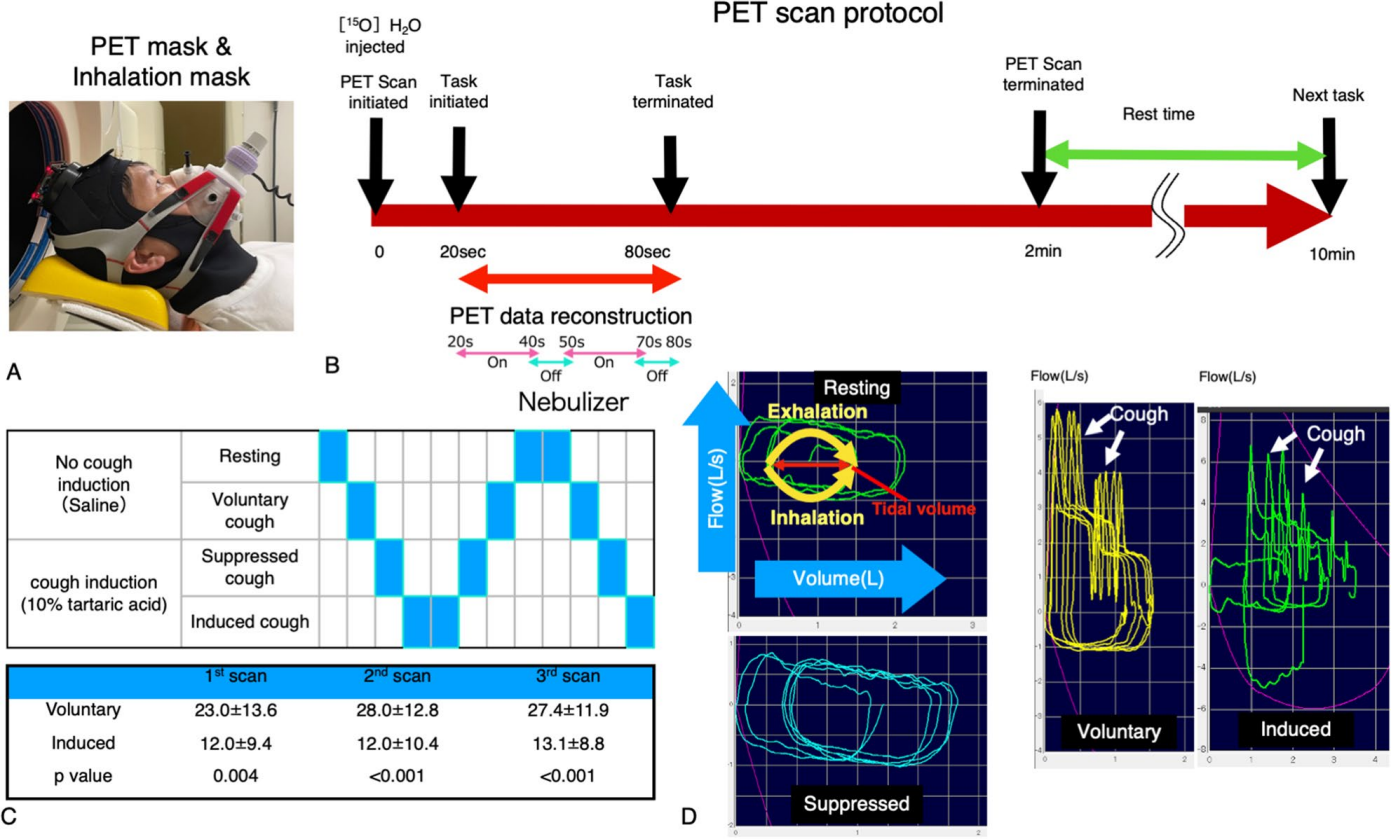
(**A**) Photograph of the head cap and inhalation mask; (**B**) PET scan protocol. (**C**) The order of the tasks was counterbalanced. The number of coughs was compared between voluntary or induced coughs (mean ± SD). (**D**) Spirometry results corresponding to each task.

The number of coughs measured during PET measurement was evaluated using Student’s t test. Significant differences were found between the VC (26.1 ± 12.8, mean ± SD) and IC groups (12.4 ± 9.4) using one-way analysis of variance (repeated-measures ANOVA). The number of coughs during the task was found to be consistent across the measurements (Fig. 1).

Comparisons of urge-to-cough scores among cough conditions were performed using the Friedman test with multiple comparisons. Significant differences were found between the cough-induced and cough-suppressed conditions relative to the resting and VC conditions (resting vs. induced, p < 0.001, resting vs. suppressed p < 0.001, induced vs. voluntary, p < 0.001 by t-statistics). The median and interquartile range for each task at baseline are as follows: resting (median 1, interquartile [1–2]), voluntary (2 [1–2]), induced (6 [4–7]), and suppressed (5 [4–7]). No difference was found between resting and voluntary conditions.

### Imaging data during PET measurement

#### Brain activation in VC vs. resting

**Figure 2:**
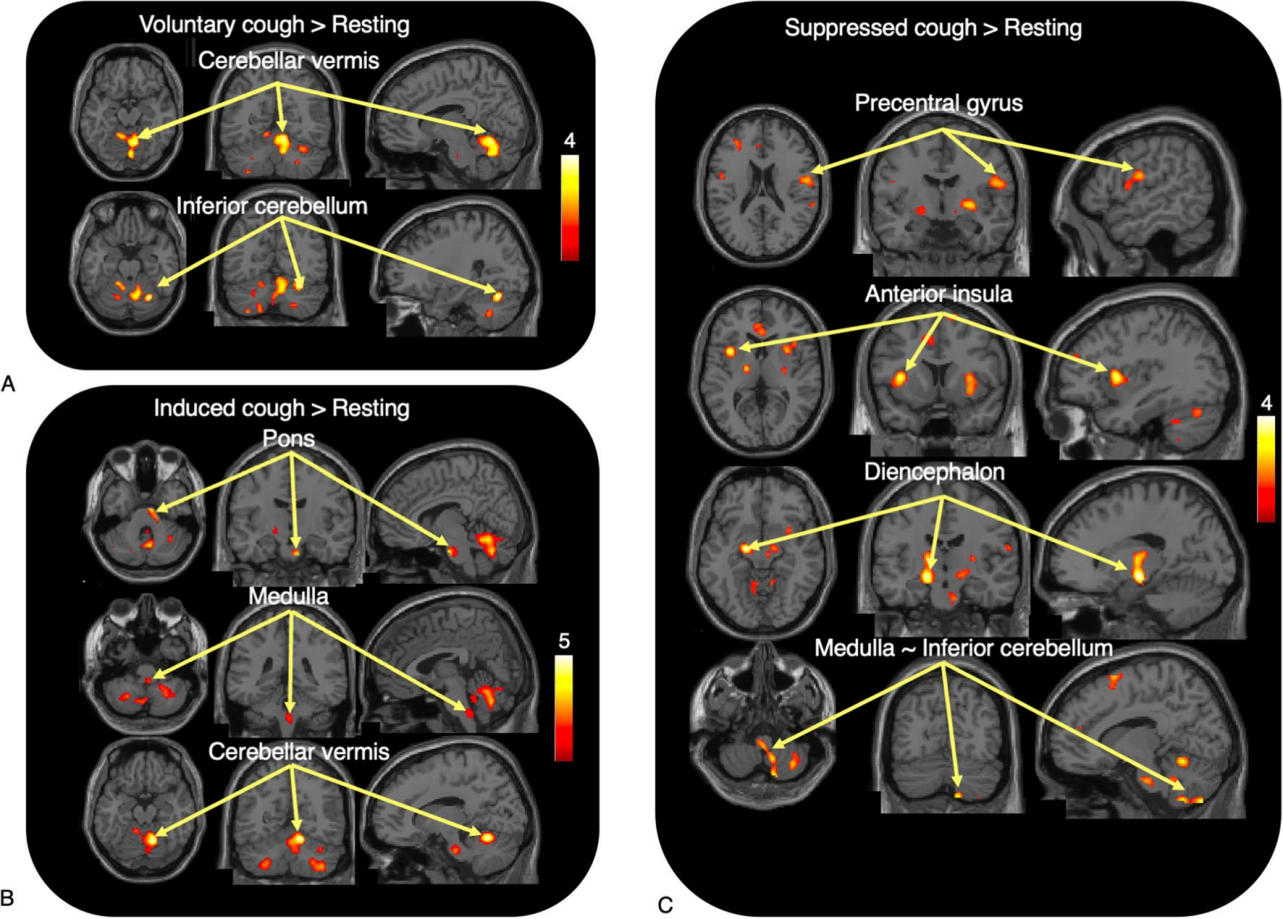
Statistical parametric mapping (SPM) results. Brain regions with significant activation in voluntary cough (**A**), induced cough (**B**) and suppressed cough (**C**) compared with rest. See Table 1 for a list of activated areas. The color bar indicates the t values.

The regions predominantly activated during the VC task were examined (Table 1, Fig. 2A). Activation of the left supplementary motor cortex was found only in the supratentorial region. In the infratentorial area, the cerebellar vermis extending to the pons and the right cerebellar hemisphere were also observed to be significantly activated. In the current analysis, we did not include the number of coughs throughout the study, as the number of coughs was zero in SC, and the factors (scan order, subject's urge to cough, PET-related global signal intensities) was considered as a covariate. In a preliminarily analysis of comparing VC to resting state by incorporating the number of coughs as a covariate, enhanced responses were observed in the bilateral supplementary motor areas (Suppl. Table 1).

**Table 1:** Brain regions with increased activation associated with voluntary, induced, and suppressed coughs.

Region	Voluntary				Induced				Suppressed				Suppressed with urge to cough			
	x	y	z	Z score	x	y	z	Z score	x	y	z	Z score	x	y	z	Z score
ACC									0	34	10	3.18	− 6	22	24	3.81
MCC									− 8	20	32	3.07				
SFC									14	10	68	3.40	− 6	28	56	3.66
MFC													− 30	30	26	3.42
													30	52	14	3.06
LOFC													− 42	42	− 10	3.40
SMA	0	4	68	3.04												
PrC									− 48	0	14	3.13	58	4	10	3.51
Caudate													20	26	− 2	2.83
Operculum													56	5	11	3.03
Putamen									30	− 2	− 2	3.59	28	− 4	− 2	3.97
Anterior insula									− 36	10	6	3.68	− 38	10	6	2.97
SPC													− 46	− 38	56	3.39
Diencephalon									− 16	− 14	− 10	4.22	− 16	− 14	− 14	4.05
Pons					8	− 12	− 32	4.11	12	− 20	− 32	3.12				
Medulla oblongata													0	− 36	− 44	2.98
Cerebellar vermis	10	− 58	− 16	3.60	14	− 58	− 18	5.49								
Inferior cerebellum	28	− 64	− 22	3.84	38	− 58	− 50	3.49	12	− 56	− 18	3.42	12	− 72	− 52	3.59
	− 32	− 60	− 54	2.98	36	− 54	− 30	3.02	34	− 58	− 48	3.25	12	− 54	− 14	3.08

The coordinates of maximally activated voxels are shown in MNI space where x, y, and z coordinates represent the left–right, posterior–anterior, and inferior–superior positions (in millimeters) from the anterior commissure. *ACC* anterior cingulate cortex, *MCC* middle cingulate cortex, *SFC* superior frontal cortex, *MFC* middle frontal cortex, *LOFC* lateral orbitofrontal cortex, *SMA* supplementary motor area, *PrC* precentral cortex, *SPC* superior parietal cortex, *CUN* cuneus, *SMC* supramarginal cortex.

#### Brain activation in IC vs. resting

While no significant responses were found in the cerebral cortical areas, the infratentorial regions, including the pons, medulla oblongata and cerebellar regions, were activated (Table 1, Fig. 2B). Among these regions, the pons and vermis had especially strong activation.

#### Brain activation in SC vs. resting

Significant activation was found in the superior frontal region covering the sensory cortex and the anterior and middle cingulate cortices. Strong activation was also found in the diencephalon, left anterior insula and right putamen (Table 1, Fig. 2C). Incorporating the parameters of the urge to cough into the analysis, additional responses appeared in the operculum and weakly in the caudate nucleus.

#### Comparisons of brain activations in IC with VC or SC

**Figure 3:**
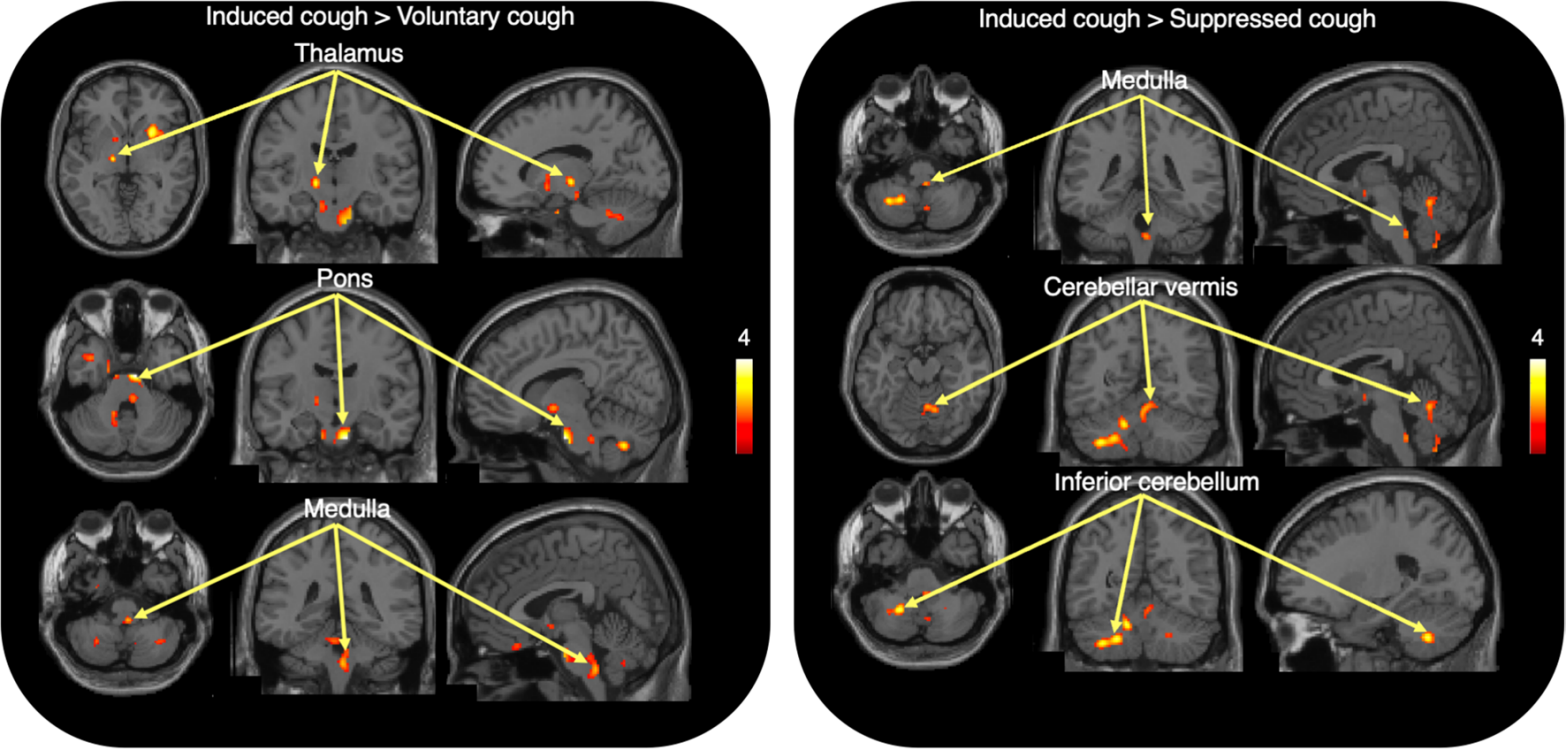
Statistical parametric mapping (SPM) results. Brain regions with significant activation in induced cough vs. voluntary cough and induced cough vs. suppressed cough. See Table 2 for a list of activated areas. The color bar indicates the t values.

Comparison of IC with VC to examine the contribution of the sensory component to cough revealed that the basal forebrain, left thalamus, pons, medulla oblongata, and cerebellum were significantly activated (Table 2, Fig. 3). Specifically, the thalamus and cerebellar vermis were found to be most strongly activated, and the putamen was also activated to some extent. Taking the urge to cough into consideration in the analysis, we found that a wider range of cerebral regions played a role in the regulation of cough under sensory stimulation. Interestingly, compared with SC, only the cerebellar regions were strongly activated, and some weak activation was observed in the medulla (Table 2, Fig. 3), which indicated that these infratentorial regions are implicated in the motor component of cough regulation.

**Table 2:** Brain regions showing higher activation in induced cough compared with voluntary and suppressed coughs.

Region	IC > VC								IC > SC							
					Covariate of urge to cough								Covariate of urge to cough			
	x	y	z	Z score	x	y	z	Z score	x	y	z	Z score	x	y	z	Z score
Basal forebrain	18	6	− 18	3.88												
MFC					0	26	− 22	3.50								
Putamen	26	12	− 2	3.54	26	14	0	3.85								
Thalamus	− 14	− 16	− 2	3.33												
Entorhinal cortex	− 24	− 6	− 40	3.37												
SMC					42	− 38	28	3.05								
Pons	10	− 12	− 32	4.19	6	− 34	− 32	3.73								
Medulla oblongata	4	− 38	− 44	2.98												
Cerebellar vermis	10	− 62	− 38	3.24					4	− 58	− 16	2.48	14	− 62	− 16	2.68
Inferior cerebellum	–				46	− 64	− 30	2.98	− 22	− 56	− 42	3.25	− 14	− 58	− 30	3.28
									− 14	− 56	− 30	3.18	− 34	− 58	− 46	3.06

The coordinates of maximally activated voxels are given in MNI space where x, y, and z coordinates represent the medial–lateral, anterior–posterior, and superior–inferior distances (in millimeters) from the anterior commissure. *ACC* anterior cingulate cortex, *MFC* middle frontal cortex, *SMC* sensorimotor cortex.

#### Brain activation by tartaric acid stimulation

**Figure 4:**
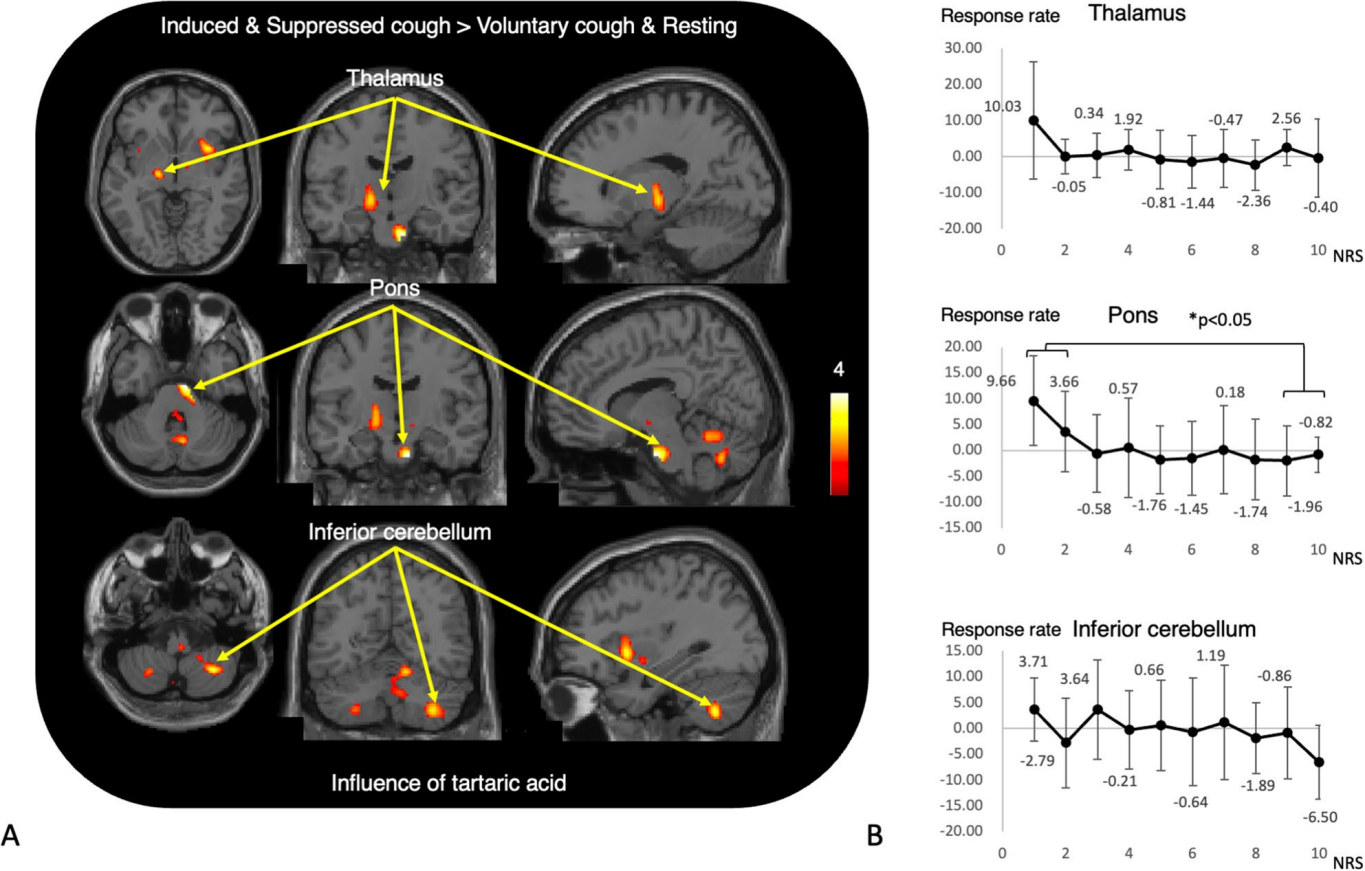
(**A**) Statistical parametric mapping (SPM) results. Brain regions significantly activated by the tartaric acid stimulus, identified by comparing the combination of induced and suppressed conditions with the combination of voluntary and rest conditions. See Table 3 for a list of activated areas. The color bar indicates the t value. (**B**) There was a significant difference between pontine responses at NRS 1–2 and those at NRS 9–10 in the baseline condition tartaric acid stimulation.

We investigated the effects of tussive agent (tartaric acid) on the brain by comparing conditions with tartaric acid (IC + SC) and without tartaric acid (VC + rest). This comparison showed significant activation in the right putamen and left thalamus in conditions with tartaric acid (Table 3, Fig. 4A). The pons and cerebellar regions were also activated during the tartaric acid–treated condition. In addition, incorporating the urge-to-cough score into the analysis showed a wider area of significant activation, including the cerebral cortex and diencephalon (Table 3). The brain response in the pons (p < 0.05) at tartaric acid stimulation was higher at NRS grades 1–2 than at NRS at grades 9–10, suggesting that the pons is not directly related to internal emotional changes (Fig. 4B).

**Table 3:** Brain regions with activation related to tartaric acid stimulation.

Region	IC + SC > VC + Resting				IC + SC > VC + Resting			
					Covariate of U to C			
	x	y	z	Z score	x	y	z	Z score
SFC					− 4	28	56	3.63
MFC					28	50	12	4.18
Putamen	32	12	− 2	3.66				
Thalamus	− 16	− 14	− 4	3.26				
Operculum	− 50	0	12	2.94	− 44	− 2	− 18	2.90
MTC					58	− 18	− 18	3.52
SMC					− 50	− 38	46	3.51
Diencephalon					− 16	− 14	− 12	3.26
Pons	10	− 12	− 32	4.52	10	− 12	− 32	3.73
Cerebellar vermis	14	− 58	18	3.31				
Inferior cerebellum	34	− 58	− 48	3.71	16	− 58	− 16	3.59
					− 24	− 80	24	2.99

The main effect of cough under evoked stimuli was determined by contrasting the presence or absence of cough events (induced cough > suppressed cough) under evoked cough. The coordinates of maximally activated voxels are given in MNI space, where x, y, and z coordinates represent the medial–lateral, anterior–posterior, and superior–inferior distances (in millimeters) from the anterior commissure. *SFC* superior frontal cortex, *MFC* middle frontal cortex, *MTC* middle temporal cortex, *SMC* supramarginal cortex.

## Discussion

The present study was the first to depict brain activity during coughing using our restraint-free PET system that enables the collection of positron signals per second while head motion was corrected every 4 ms. Throughout this study, cerebellar activation was highlighted during cough irrespective of voluntary and induced conditions, while brain activity in the cerebral cortex was found in more limited conditions. Indeed, suppressing cough activated regions such as the cingulate cortex, central posterior gyrus, left insular cortex and left diencephalon. In the present study, we used tartaric acid to induce cough. As reported before^7^, there were significant differences in the urge to cough and the number of coughs during the cough-inducing stimulus compared to placebo. Although participants were completely blinded to the administration of tartaric acid, some subjects felt a strong urge to cough even when saline was administered.

Using the current head motion correction method, we found that brain activity during the coughing task was much more localized than previously reported, as we had hypothesized prior to this study. Brain activity during cough has been thought to be controlled in a top-down manner from higher brain regions via the network stimulated by brainstem input^8,9,11^. Comparing brain activity at rest and during other tasks, significant brain activation during VC was chiefly confined to the cerebellum extending to the pons. In contrast, during IC, significant activation was found in the diencephalon to the brainstem, including the hypothalamus and cerebellum (Table 1, Fig. 2B). These findings indicate that the cerebellum is a key player in regulation of cough.

The cerebellum is involved in coordinating the timing of lingual and oral motor movements during swallowing^18^. Stimulation of the cerebellum with rTMS has been reported to activate areas of the cerebral cortex involved in swallowing movements. A study examining coughing behavior in cats after cerebellectomy did not reach a conclusion that the cerebellum is tightly associated with coughing, in which muscle activity during cough increased immediately after cerebellectomy and its movement during cough varied randomly over time^19^. Cerebellar activity in the present study was still observed after head motion correction, suggesting that the cerebellum may contribute to the coordination and planning of coughing movements in the same way that it functions during eating and swallowing.

Suppressing cough is an act of motor inhibition. It has been reported that suppressing a cough activates the dorsomedial prefrontal cortex, middle cingulate cortex, supplementary motor cortex, right lateral inferior frontal gyrus, caudate nucleus, and right insular cortex^9,11,20^, suggesting the existence of an inhibitory network for coughing. Within this network, the anterior cingulate cortex, insula, and midbrain are also activated by painful stimuli, suggesting a link to the suppression of cough^21–23^. It was reported that nociception and cough show similar peripheral and central mechanisms^24–27^. Indeed, the regions associated with cough suppression in the present study were similar to those reported above. Furthermore, we found a response that was even more consistent with previous studies after we incorporated the intensity of the urge to cough into the analysis. As hypothesized, brain activations in the caudate nucleus, operculum, and infratentorial brain regions (cerebellum to medulla oblongata) might occur as a result of a stronger perception of the cough impulse. The fact that cerebellar activation was still observed during cough suppression suggests that cerebellar control may be important in the inhibition of cough generation.

Reflex cough is mediated by vagal afferents from the upper airways and the tracheobronchial tree^28–30^. In the present study, the brainstem was activated in the IC vs. VC and SC vs. rest conditions when the urge to cough incorporated (Fig. 1, Table 1). Both VC and the cough reflex are thought to involve the brainstem. In particular, the medulla oblongata responds to the induction of cough and exhibits different activation depending on the type of coughing^9,12^. This may indicate that the brainstem is not strongly involved in voluntary (more precisely, “automatic”) cough in the present study. The sensory processing of cough is considered to involve the pathways of the solitary nucleus and the paratrigeminal nucleus in the medulla oblongata^30–33^. In the present study, activation in the brainstem during cough induction occurred in the brainstem covering the solitary nucleus, the dorsal nucleus of the vagus nerve, and the hypoglossal nucleus in the medulla oblongata during cough induction. The solitary nucleus projects Aδ fibers that are distributed in the trachea and larynx^30,34^. In contrast, the paratrigeminal nucleus, which corresponds to the C fibers distributed in the nociceptors of the lung^35^, was not significantly activated. Therefore, stimulation at the pharyngeal level by cough medicines can effectively induce coughing.

**Figure 5:**
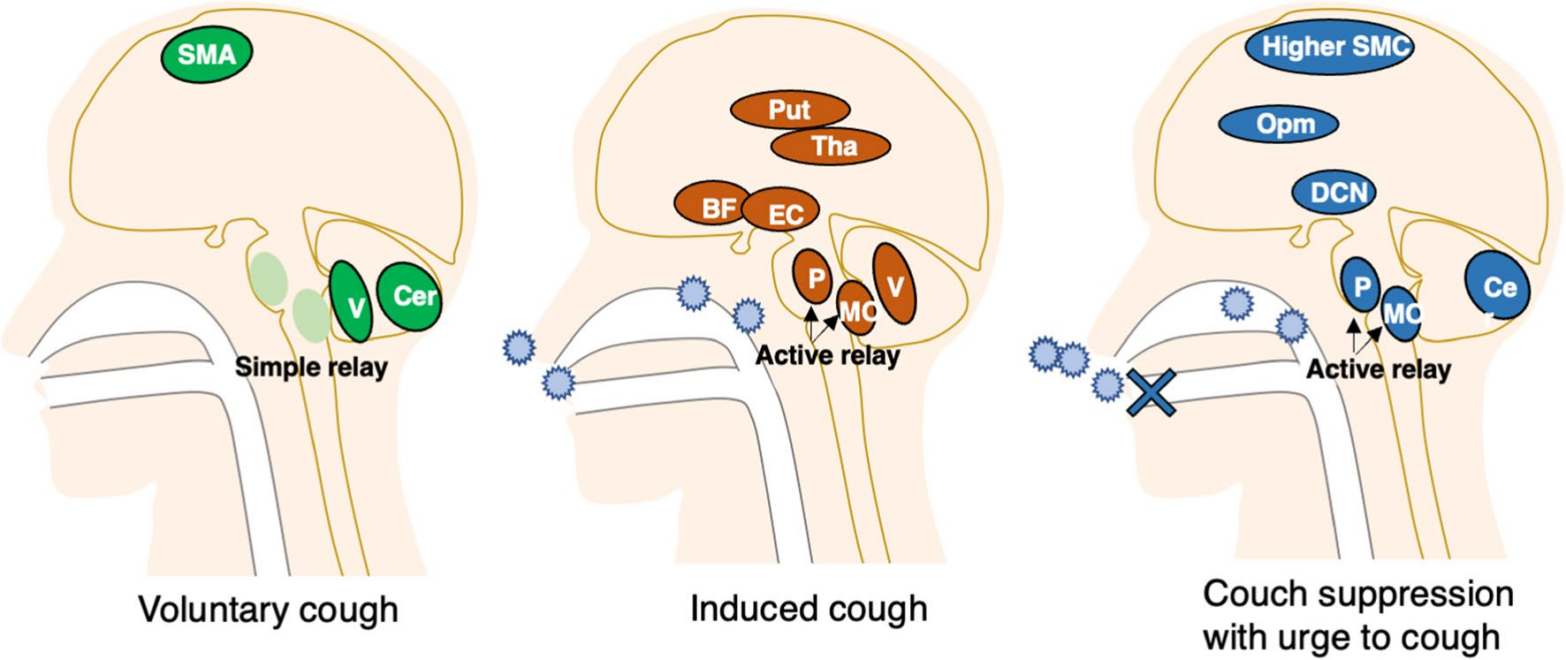
A hypothesized schematic brain network for cough. Brain regions that significantly contribute to cough regulation in each cough condition are illustrated with different colors. The pons and brainstem may be the most important regions in the control of stimulated cough. *SMA* supplemental motor area, *V* cerebellar vermis, *Cer* cerebellar hemisphere, *Put* putamen, *Tha* thalamus, *BF* basal forebrain, *EC* entorhinal cortex, *P* pons, *MO* medulla oblongata, *SMC* sensorimotor cortex, *Opm* operculum, *DCN* diencephalon.

In our daily clinical practice, we encourage dysphagic patients to promote voluntary coughing and occasionally use CiTA to induce coughing. We compared brain activity during induced coughing by the CiTA protocol with voluntary or suppressed coughing to examine the sensory (IC > VC) and motor (IC > SC) components of the cough. In IC compared with SC, the motor component showed responses in the cerebellum, excluding the vermis. The current analyses suggest that the cerebellar vermis might be associated with the sensory control component (Table 2, Figs. 3, 5), whereas the lateral regions of the cerebellar hemispheres might be important in the motor control component (Table 2, Figs. 3, 5). Brain responses in the pons and cerebellar hemispheres may be related to coughing behavior itself, irrespective of the internal urge to cough (Figs. 4B, 5). It has been reported that both motor and sensory components related to coughing are derived from networks involving extensive brain activity^9^. The activated regions in both components in the present study were narrower than the range reported before. An animal experiment with rats showed that cough-inducing stimuli activated a pathway between the brainstem and the subthalamic nucleus and that the input of nociceptive stimuli to the brainstem is under descending control of central origin. The present results highlighted the forebrain, thalamus, and brainstem activity as the basis of the sensory component. As suggested elsewhere, it is likely that the input from induced stimuli is perceived in the diencephalon and brainstem, while the basal forebrain and thalamus are involved in making adjustments to the sensory input^36^. It was reported that the forebrain network was less active during cough suppression in patients with chronic cough (persistent natural induction of cough), while it was more active in healthy subjects^20^. The present response in the healthy subjects was compatible with this observation. Based on previous literature, we also made comparisons between tasks with CiTA and tasks without CiTA to examine the effect of tartaric acid in the context of cough. This comparison showed significant brain activation in the putamen, thalamus, operculum, pons, and cerebellum (Fig. 5, Table 3), suggesting that CiTA may be an effective tool to activate the sensory and perceptual control system. With the urge to cough incorporated, broader regions in the cerebral cortex were included as regions with significant activation in the CiTA-related condition, possibly because the dorsomedial prefrontal cortex became responsive when the coughing impulse component was added. In the current study, the tussive agent was continuously delivered in short suspensions (10 s) for a total of 40 s to induce a sufficient number of coughs while avoiding respiratory stress. In preliminary experiments, stress was frequently observed due to tidal respiration. So, the outcomes might have been different if the tidal breathing of tussive agent was introduced.

The limitations of this study are as follows. Current cough tasks do not allow clear separation of cough components in terms of central nervous system mechanisms. While VC and IC may be motor- and sensory-dominant, respectively, the presence of cough and reflex impulses cannot be completely excluded. The subjects were instructed to sit on a reclining chair while coughing but not to hit their head against the internal wall of the gantry. This indicates that cough in the current situation differs from cough in the real-world situation. Although we used 10% tartaric acid, similar to the concentration used at the bedside, the content was not optimized for each participant. Therefore, the number and magnitude of coughs varied depending on their responsiveness. It may be necessary to set the concentration of cough-inducing substances administered based on the guidelines of the European Respiratory Society (ERS). Additionally, respiratory status during coughing may be a potential confounding factor, as respiratory rhythm can influence emotion and cognition in brain physiology^37^. This may be a common black box in brain mapping studies. The current study was exploratory in nature and considered the whole brain, including the brainstem, to be part of search area; accordingly, the intensity and extent of the signal were thresholded without correction for multiple comparisons. In addition, this whole-brain analysis is not ideal for exploring the specific areas of activation within the brainstem because PET has lower spatial resolution than fMRI.

In conclusion, the cerebellum plays a role in the regulation of cough not only through motor coordination but also through the perceptual control of cough. The brainstem is especially involved in the manipulation of cough (Fig. 5). We discuss our future outlook. The current study partly clarified the underlying mechanism of CiTA, which allows clinicians to predict whether it will be effective before attempting it in patients with brain lesions who have sustained damage to the regions related to cough control. To confirm this, the current study is worth extending into a future study in dysphagic patients.

## Materials and methods

### Participants

Twenty-four healthy male volunteers (27.4 ± 4.0) were recruited. Male-only participation was adopted because in women there were confounding effects such as hormonal fluctuations and pregnancy. All participants were right-handed nonsmokers who drank occasionally; had no neuropsychiatric, respiratory or allergic disorders; and did not use medication that could affect cognitive function. Prior to participation, the subjects underwent respiratory function tests using a spirometer (Minato Medical Science Co., Ltd., Osaka, Japan) to confirm that they had no lung function impairment, and they inhaled a 10% solution of l-tartaric acid with a nebulizer (Shinei Kogyo Co., Ltd., Saitama, Japan) to assess whether their cough reflex was adequate for the planned study. Participants were excluded if no cough reflex was generated within 30 s after nebulizer inhalation. Data from one participant were excluded because he had difficulty completing all scans due to a sensation of fatigue. The study was conducted from September 2021 to July 2022 in compliance with the Declaration of Helsinki and with the approval of the clinical ethics committees of Hamamatsu University School of Medicine [20-328], Hamamatsu City Rehabilitation Hospital [21-09], and Hamamatsu Photonics K.K. (H-184). Written informed consent was obtained from all participants.

#### MRI data acquisition

We performed MRI to exclude brain abnormalities and to conduct anatomic normalization using an Optima MR360 Advance 1.5 T scanner (GE Healthcare) with a 12 channel head coil. Structural T1-weighted images were acquired in the sagittal plane (124 slices, 1.4 mm slice thickness, 0.98 × 0.98 mm^2^ in-phase resolution, echo time (TE) 5.1 ms, repetition time (TR) 12.2 ms, flip angle 25°) in 5 min 0.4 s of scanning time.

### PET data acquisition and tasks

We used a brain PET system (HITS655000: Hamamatsu Photonics K.K., Hamamatsu, Japan)^16^. [^15^O]H_2_O was used to evaluate brain activity with the head unrestrained. Participants were fitted with masks for cough measurement (Fig. 1). The mask was connected to the previously mentioned spirometer and nebulizer.

[^15^O]H_2_O experiments consisted of four tasks: resting, VC (simple repetition of cough), IC (using CiTA) and SC (suppressing cough against stimulation by CiTA). In the VC condition, subjects were instructed to cough as much as possible within their ability. Due to the counterbalance of tasks performed, the number of coughs in the VC might vary as seen in Fig. 1C. For CiTA, a 10% solution of l-tartaric acid was used in the IC and SC tasks, and saline was used as a placebo during the resting and VC tasks. No information about task content was given during the study. These sessions were counterbalanced across the study. The route for [^15^O]H_2_O administration was secured in the right forearm. Two-minute PET scans were performed after tracer injection (list-mode data acquired every second). The task was initiated 20 s after tracer injection and continued for one minute, in which the timing of tracer entry into the brain was optimally determined during the task. The tussive agent (tartaric acid) was delivered continuously through the mask connected with the nebulizer via a nylon tube. The nebulizer was turned on for 20 s and off for 10 s for a total of two cycles to avoid subject stress due to continuous inhalation of tartaric acid (Fig. 1). We used 60 s of [^15^O]H_2_O data reconstructed after the increase in the PET count of the brain. The dose of [^15^O]H_2_O injected was 2.5 MBq/kg per scan. During the [^15^O]H_2_O experiment, the number of coughs and the intensity of coughing (cough peak flow) were measured (see the waveforms measured by spirometry in Fig. 1). At the end of each scan, the urge to cough during the task was checked on a 10-point scale using the numerical rating scale (NRS). For each task, those indices were included in the analysis.

### PET data reconstruction

During the PET scans, the participants wore a head cap with four LED markers. The locations of these markers were tracked every 4 ms by two high-speed cameras (HAMAMATSU Intelligent Vision System, IVS) mounted at the back of the gantry. Using information about head movements monitored during the scans, we corrected the line of response (LOR) of list-mode data and reconstructed the image using a 3D list-mode dynamic row-action maximum likelihood algorithm (DRAMA) by applying a quaternion algorithm to variations of LED markers. The accuracy of this method was reported in detail in our previous paper using phantom studies using the quaternion formula^17^. The PET images were reconstructed with a matrix of 128 × 128 × 42 voxels (voxel size: 2.6 × 2.6 × 3.4 mm^3^). Images were corrected for attenuation and scattered events with emission-based segmentation attenuation correction (ESAC) and single scatter stimulation (SSS), respectively. For the image reconstruction of each 2-min [^15^O]H_2_O PET scan, we selected 60-s time frames that started 10 kcps after the onset of the scan. Head movements were corrected every 4 ms, whereas PET data were acquired every 1 s. Since head movements within 4 ms can be accurately corrected, it is possible that the movement and image inhomogeneity have a combined effect. However, statistical procedures involving image normalization and smoothing of images minimize such combined effects on brain mapping at the macroscopic level.

### PET data analyses

We performed whole brain analyses of [^15^O]H_2_O PET data with Statistical Parametric Mapping software (SPM12; Wellcome Trust Center for Neuroimaging, London, UK) running on MATLAB software (MathWorks, Natick, MA). We realigned the reconstructed [^15^O]H_2_O PET images to the mean image of each participant. Structural images of each participant were coregistered to the mean image of the realigned [^15^O]H_2_O PET volumes. The coregistered structural images were spatially normalized to the standard brain space as defined by the Montreal Neurological Institute (MNI) using the unified segmentation algorithm with light regularization, which is a generative model that combines tissue segmentation, bias correction, and spatial normalization in the inversion of a single unified model^38^. After spatial normalization, the resultant deformation field was applied to the realigned [^15^O]H_2_O PET imaging data, which were resampled every 2 mm using seventh-degree B-spline interpolation. All normalized functional images were then smoothed using an isotropic Gaussian kernel of 8 mm full width at half maximum (FWHM). We assumed that the scan-to-scan variability within a PET session and the session-by-contrast interactions were approximately equal in our [^15^O]H_2_O PET measurements. We thus adopted a fixed-effect analysis with a multiparticipant and multi-condition full factorial design and included all scans of all participants into a single general linear model in SPM. Condition-specific effects were estimated with a general linear model, whereas the order of PET image acquisition, subjects’ urge to cough, and global signal intensities were used as covariates in the design matrix to account for the effect of these variables and global signal normalization. The motion parameters as well as the order of PET image acquisition was treated as a covariate so that the influence of the body motion would not be all or nothing and would be affected by time. Comparison of adjusted mean reginal cerebral blood flow between conditions was performed on a voxel-by-voxel basis with t-statistics. The resultant set of voxel values for each contrast constituted a t-statistic SPM{t}, which was then transformed to a unit normal distribution (SPM{z}) map^39^. The current whole brain statistical maps were thresholded at a peak level of p < 0.001 (Z value > 2.97) without correction for multiple comparisons in the cluster-level p value because of the exploratory nature of this study.

### Supplementary Information

Supplementary Table 1.

## Data Availability

The datasets generated and/or analyzed during the current study are not publicly available due to the absence of agreement from the participants but are available from the corresponding authors on reasonable request.
