# Supplementary material for: Neural substrates of cough control during coughing
Source: Sci Rep. 2024 Jan 8;14:758. doi: 10.1038/s41598-024-51477-x (PMC10774348; doi:10.1038/s41598-024-51477-x)
Supplement: Supplementary file 1 — Supplementary Table 1. [file 41598_2024_51477_MOESM1_ESM.pdf]

Title: Neural substrates of cough control during coughing

Authors

Takafumi Sugi[1,2], Tomoo Inubushi[3], Tomohisa Ohno[4], Yuya Onishi[3], Takashi Isobe[3], Takashi Shigematsu[2], Satoshi Hanai[5], Yoshiro Okada[5], Ryosuke Takahashi[5], Yuichi Tawara[6], Chie Suzuki[7], Toshihiko Kanno[8], Yasuhiro Magata[7], Ichiro Fujishima[2], Etsuji Yoshikawa[3], Yasuomi Ouchi[1,8]

1. Department of Biofunctional Imaging, Hamamatsu University School of Medicine, 1-20-1, Handayama, Higashi-Ku, Hamamatsu, Shizuoka 431-3192, Japan
2. Department of Rehabilitation Medicine, Hamamatsu City Rehabilitation Hospital, 1-6-1 Wagokita, Naka-ku, Hamamatsu, Shizuoka 433-8511, Japan
3. Central Research Laboratory, Hamamatsu Photonics K.K., 5000, Hirakuchi, Hamakita-Ku, Hamamatsu, Shizuoka 434-8601, Japan
4. Department of Dentistry, Hamamatsu City Rehabilitation Hospital, Hamamatsu, Japan
5. Department of Rehabilitation, Hamamatsu City Rehabilitation Hospital, Hamamatsu, Japan
6. School of Rehabilitation Sciences, Seirei Christopher University, 3453, Mikatahara Kita-ku, Hamamatsu,Shizuoka, 433-8105, Japan
7. Department of Molecular Imaging, Hamamatsu University School of Medicine, 1-20-1 Handayama, Higashi-ku, Hamamatsu, Shizuoka, 431-3192, Japan
8. Hamamatsu Medical Imaging Center, Hamamatsu Medical Photonics Foundation, Shizuoka 434-0041, Japan

Supplementary1.: Brain regions with increased activation associated with voluntary with the number of coughing

	Voluntary with number of cough			
Region	x	y	z	Z score
SMA	-2	-6	72	3.28
MTC	-36	6	-46	3.24
Lingual gyrus	4	-82	-12	3.09
Mid brain	-2	-22	-12	2.97
Inferior cerebellum	26	-50	-40	4.01
	-30	-60	-54	3.93
	40	-46	-52	3.30
	-44	-54	-44	3.28

The coordinates of maximally activated voxels are shown in MNI space where x, y, and z coordinates represent the left-right, posterior-anterior, and inferior- superior positions (in millimeters) from the anterior commissure. SMA: supplementary motor area, MTC: middle temporal cortex
